# Visual Exploratory Search of Relationship Graphs on Smartphones

**DOI:** 10.1371/journal.pone.0079379

**Published:** 2013-11-04

**Authors:** Jianquan Ouyang, Hao Zheng, Fanbin Kong, Tianming Liu

**Affiliations:** 1 College of Information Engineering, Xiangtan University, Xiangtan, Hunan, China; 2 Department of Food Science, the University of Georgia, Athens, Georgia, United States of America; 3 Department of Computer Science, the University of Georgia, Athens, Georgia, United States of America; University of Bath, United Kingdom

## Abstract

This paper presents a novel framework for Visual Exploratory Search of Relationship Graphs on Smartphones (VESRGS) that is composed of three major components: inference and representation of semantic relationship graphs on the Web via meta-search, visual exploratory search of relationship graphs through both querying and browsing strategies, and human-computer interactions via the multi-touch interface and mobile Internet on smartphones. In comparison with traditional lookup search methodologies, the proposed VESRGS system is characterized with the following perceived advantages. 1) It infers rich semantic relationships between the querying keywords and other related concepts from large-scale meta-search results from Google, Yahoo! and Bing search engines, and represents semantic relationships via graphs; 2) the exploratory search approach empowers users to naturally and effectively explore, adventure and discover knowledge in a rich information world of interlinked relationship graphs in a personalized fashion; 3) it effectively takes the advantages of smartphones’ user-friendly interfaces and ubiquitous Internet connection and portability. Our extensive experimental results have demonstrated that the VESRGS framework can significantly improve the users’ capability of seeking the most relevant relationship information to their own specific needs. We envision that the VESRGS framework can be a starting point for future exploration of novel, effective search strategies in the mobile Internet era.

## Introduction

Traditional lookup mode of human-computer interaction in Web search has been very successful in presenting search results to the user in an ordered list in relation to some measurement of relevance to the query, e.g., in popular commercial search engines such as Google, Yahoo! and Bing. Recently, the exploratory search approach has received increasing interest in the literature [1-9] as this methodology enables users to explore, adventure, and discover in a rich information world. This overall trend of involving more active user engagement in the search process is driven by the explosion of Web data and knowledge, technical need of selection, navigation, and trial-and-error tactics during web browsing, and the intrinsic user need of information/knowledge seeking and online learning [1]. These visual exploratory search approaches have been empowered by recent methodological advancements in information retrieval [10-16], human-computer interaction [10,17,18], information visualization [17,19], and knowledge engineering [20-31].

In general, searching to learn and discover has been increasingly important as mountains of data become available online. Unfortunately, current available search engines can help very little. For instance, Web users have to face a common problem nowadays: the information on the Web is not too little, but too much. For a typical keyword(s) query, current search engines such as Google, Yahoo! or Bing will most likely return many result pages or documents that are far beyond the users’ capability of full comprehension and understanding. More importantly, the relationships among the large amount of returned results by these common search engines are unclear, and thus search users have to mentally figure out the semantic relationships embedded in those enormous numbers of returns with tremendous efforts. 

In comparison, visual exploratory search typically entails multiple iterations and return sets that require the user’s cognitive processing and interpretation [1]. These return items may be instantiated in various media types such as graphs and texts and often require the web searchers to navigate, view, compare, and make qualitative/quantitative judgments [1,12]. A general principle of the human memory is that it is typically much easier to recognize a keyword or name than it is to think up that keyword [32]. Thus in many application scenarios, it is very useful to prompt the searchers with information related to their information need [32]. Browsable information structures, such as the relationship graphs proposed in this paper, can give an overview of the content of interest, allowing the searcher to navigate throughout the information of interest from coarser to finer scales. Therefore, the graph-based representation of relationship information on-demand can significantly facilitate the user’s experience of search to learn and discover.

However, current representation of Web information and knowledge, e.g., by current common search engines, does not allow the abovementioned graph-based visual exploratory search for learning and discovery. Therefore, in this paper, we propose to infer relationship graphs via large-scale meta-searches [33-35] from popular common search engines, which will be re-organized and re-structured into semantic relationship networks via effective natural language processing approaches. The basic premise here is that a semantic network can be represented by relationships between concepts/terms. For example, the WordNet labels the semantic relations among natural language words [20,21]. Hence, we construct the relationship graphs of concepts/terms based on their probabilities of co-occurrences in many returned web pages or documents from the meta-searches of common search engines. A major advantage of the graph-based relationship representation is that the graphs allow users to naturally and flexibly browse and navigate throughout the graph representation of relationships in a multi-scale fashion, thus facilitating the abstraction and extraction of information at the desired levels and granularities. In addition, the graph-based relationship representation facilitates the effective integration of personalized user profiles into the whole navigation and search processes, e.g., the graph-based user profile can be matched with the graph of interest (GOI). The personalization of search is then transformed into a graph matching problem. Then, several graph theory approaches are readily available for these problems, such as multi-scale graph representation [36] and graph matching [37-39]. 

Furthermore, the proposed graph-based visual exploratory search is particularly suitable for mobile search on smartphones (such as Android and iPhone). Now, we are witnessing the explosion of mobile content and applications on smartphones. From our perspective, smartphones possess two prominent advantages for visual exploratory search of relationship graphs as follows. First, smartphones have very user-friendly interface, which partly explains their unparalleled popularities. Their touch-screen displays use state-of-the-art multi-touch technology that is capable of simultaneously monitoring two or more distinct positions of input touches. This natural and user-friendly interface is a very attractive feature for its applications in visual exploratory search. Second, smartphones have ubiquitous Internet connection and portability. The smartphones take the advantage of seamless integration of Wi-Fi and 3G wireless, offering very fast data access anytime, anywhere. Therefore, smartphones provide users with the flexibility and ability to search to learn and discover anytime, anywhere. 

In general, the proposed VESRGS framework effectively and simultaneously addresses three fundamental needs from users: visual exploratory search to learn and discover from mountains of data for their personalized needs, integration and condensation of large number of meta-search results from common search engines into relationship graphs of semantic networks, and user-friendly effective interfaces and ubiquitous availability and portability for visual exploratory search. The abovementioned three points are the major methodological contributions of this paper. The rest of the paper is organized as follows. We first survey existing approaches in exploratory search, relationship graph construction, and mobile search. The VESRGS framework is introduced in the next section, and experimental results are presented in afterwards. The last section discusses future directions of improvements and concludes this paper.

### Related works

In atypical Web search via common search engines such as Google, Yahoo! and Bing, users submit a keyword query via a search text box and receive a textual list of results. Recently, a new school of search methods called exploratory search [1,12] has emerged, which supports the exploration, learning and discovery of knowledge via a combination of querying and browsing strategies. A seminal article in [1] summarized three types of search: lookup, learn and investigate. Lookup searches are considered as traditional search, while exploratory searches relate to discovery-oriented tasks. To support exploratory search, the information retrieval community is increasingly collaborating with the human-computer interaction community to create new ways of bringing users more actively into the search processes. In the literature, several exploratory search prototype systems have been proposed. For instance, Yee et al. [13], developed an alternative interface for exploring large collections of images using hierarchical faceted metadata and dynamically-generated query previews. Alonso et al. [14], presented a novel interface that utilized timeline data to enable effective presentation and navigation of search results. Tvaroek and Bielikov [15] described a personalized faceted browser that facilitated exploratory search by offering users with an integrated search and navigation interface. 

However, these abovementioned exploratory search prototype systems were not developed specifically for mobile devices such as smartphones. Recently, there has been increasing interest in mobile search [16,18,40,41,42]. Despite helpful efforts from the community, current research on mobile search still has a number of limitations in methodologies. In a recent article [41], the authors explored the usages and visions of mobile search with users' interview-based qualitative study and had the following conclusions. First, mobile users ask for accessing the entire Internet with their mobile devices, rather than a subsection of it. Second, search success is measured based on new added-value applications that exploit unique mobile functionalities [41]. Here we believe that user-friendly multi-touch interface is a major part of the unique mobile capabilities and should be integrated in the exploratory search process. Importantly, the authors in [41] interpreted that the mobile logic should involve the use of personalized and context-based services [41]. Hence, we hypothesize that visual exploratory search of relationship graphs on smartphones will effectively fit the users’ needs, while sufficiently exploiting the full advantages of unique mobile functionalities such as user-friendly interface and ubiquitous availability and portability. 

Automatic construction of semantic networks or graphs of relationships from textual corpora has been a well-established field in the natural language processing field. Current approaches can be broadly classified into the following four general categories [22]. The first class of methods is based on distributional properties of words: it consists in studying co-occurrence distributions of terms or concepts in order to calculate a semantic distance between the concepts/terms [23-26]. The method used in this paper belongs to this category. The second school of methods employed natural language processing techniques such as pattern extraction and matching. These methods rely on the lexical or lexico-semantic patterns to identify ontological and non-taxonomic relationships between concepts in unrestricted text [27,28]. The third school of methods includes those based on dictionary definitions analysis [29], which takes advantage of the particular structure of dictionaries in order to extract relationships with which to arrange the concepts in an ontology. The fourth category of methods is the semantic network [10,30,31] based on similar nets of interdependent concepts. The dependencies can be classified into distinct types with specific interpretations. The motivation underlying semantic networks is that concepts have their meanings through their semantic relations with other concepts.

## Methods

**Figure 1:**
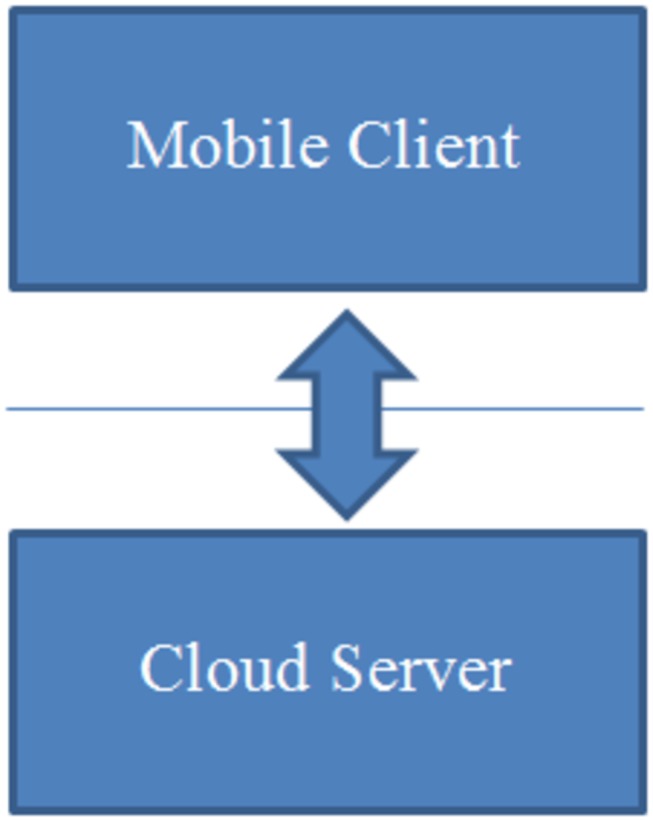
Overview of the VESRGS framework.

The overview of the VESRGS framework is outlined in Figure 1. The mobile client side of the VESRGS system allows users to visually and interactively explore and discover within the relationship graphs based on specific starting keywords, while the cloud server constructs and returns relationship graphs via meta-search and knowledge integration. The VESRGS framework is composed of three major components: inference and representation of semantic relationship graphs on the Web via meta-search, visual exploratory search of relationship graphs through both querying and browsing strategies, and human-computer interactions via the multi-touch interface and mobile Internet on smartphones. 

### Inference of semantic relationship graphs via meta-search

**Figure 2:**
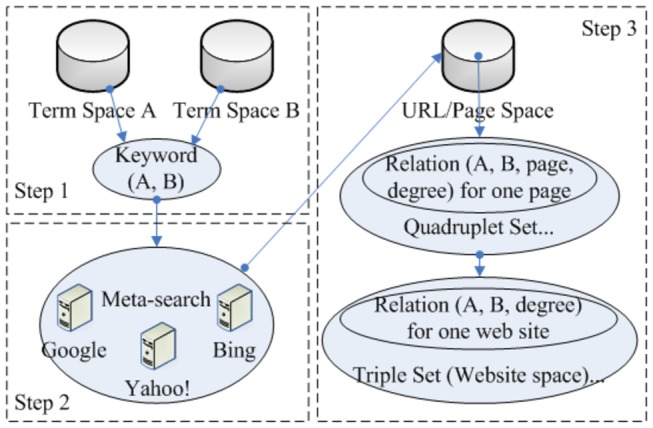
The flowchart of inferring semantic relationship graphs via meta-searches.

As illustrated in Figure 2, the construction of semantic relationship graphs is composed of three major steps: definition of two spaces of relevant keywords/terms, meta-search via popular commercial search engines, and inference of relationship graphs within the returned web pages and websites. The details of each step will be provided in the following paragraphs. 

As mentioned in the introduction, the VESRGS framework focuses on visual exploratory search of semantic graphs, which is defined as the relationships among specific terms. Without the loss of generality, here we use a specific real-world example as a test-bed to demonstrate how we construct the relationship graphs: the relationships among food and human diseases/conditions. For instance, we obtained a list of foods from the USDA website [43] and a list of human diseases from the NIH website [44]. Therefore, the two lists of foods and human diseases correspond to the term space A and B, respectively. Then, we can construct a relationship space of keyword pairs (A, B) that covers all possible combinations of terms in both spaces, as illustrated in Step 1 of Figure 2. In our experiments, the number of terms in both spaces could range from dozens to dozens of thousands, depending on specific application scenarios. Consequently, the term relationship space of keyword (A, B) could contain many pairs of terms. 

In the second step, all of the possible term pairs obtained in the first step will be sent to an in-house meta-search engine that interacts with three popular common search engines including Google, Yahoo! and Bing, as demonstrated in Figure 2. Specifically, our meta-search engine calls APIs provided by these three common search engines by inputting the keyword pairs and retrieving the return links and web pages. Due to the limits on the number of sent terms imposed by these commercial search engines, we have to send our meta-searches periodically to these three search engines. 

**Figure 3:**
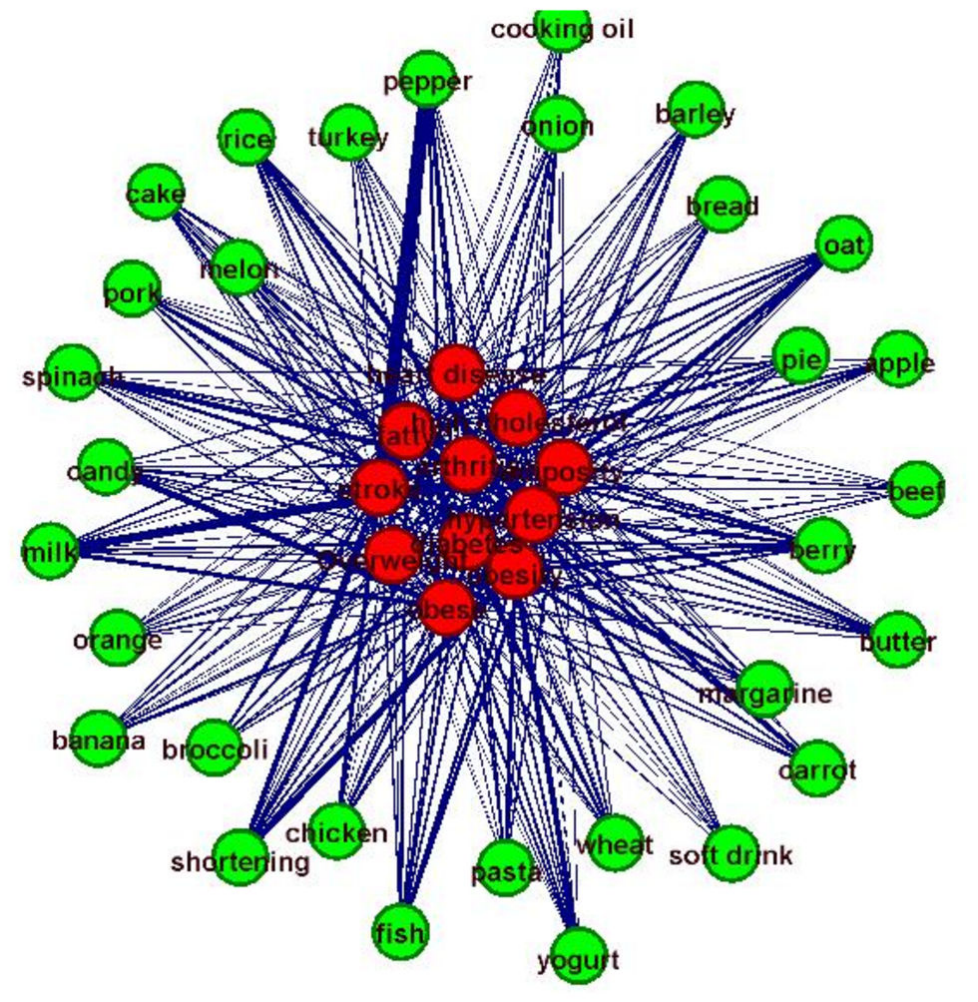
Example of constructed food/health relationship graph.

In the third step, we perform effective statistical natural language processing on the returned web pages and websites, and construct semantic relationship graphs. Specifically, for each pair of terms, we measured their probabilities of co-occurrences in all of the returned web pages from the meta-search results, which were used to quantify the semantic relationship strength of these terms. As a result, in the constructed relationship graph, the nodes are represented by the terms in two input spaces, and the edges are defined by their semantic relationship strengths. Figure 3 shows an example of the constructed semantic relationship graph. Here, the green-colored nodes are terms from the food list, while the nodes in red are extracted from the human disease/condition space. In particular, the lists of returned web pages are indexed along the graph edges. 

**Figure 4:**
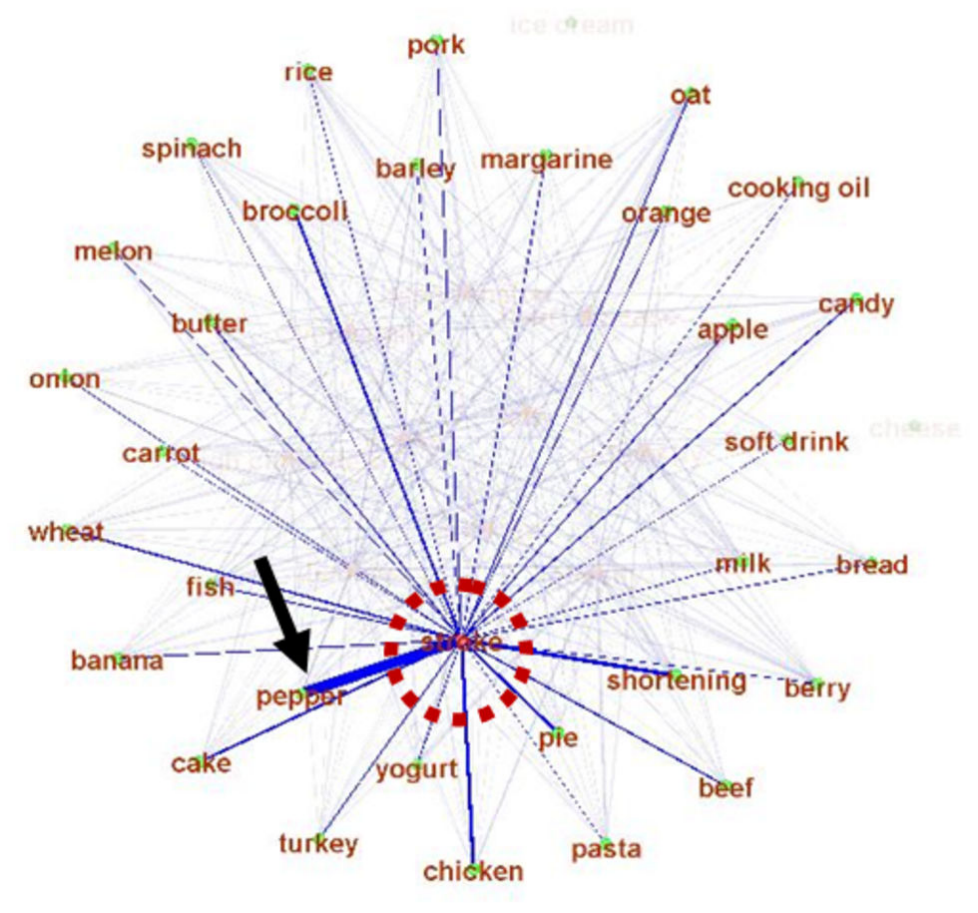
An example of the relationship between stroke (red dashed circle) and foods. The edge width represents strength.

A specific example of the inferred graph of the semantic relationship between stroke and other foods is shown in Figure 4. It shows that pepper and stroke are closely associated. When we searched the literature, it was found that proper use of pepper can promote health [45,46]. Though this association is to be further confirmed by biomedical research in the future, the close association between pepper and stroke is widely reported (the strong connection highlighted by black arrow in Figure 4). 

### Visual exploratory search of relationship graphs

**Figure 5:**
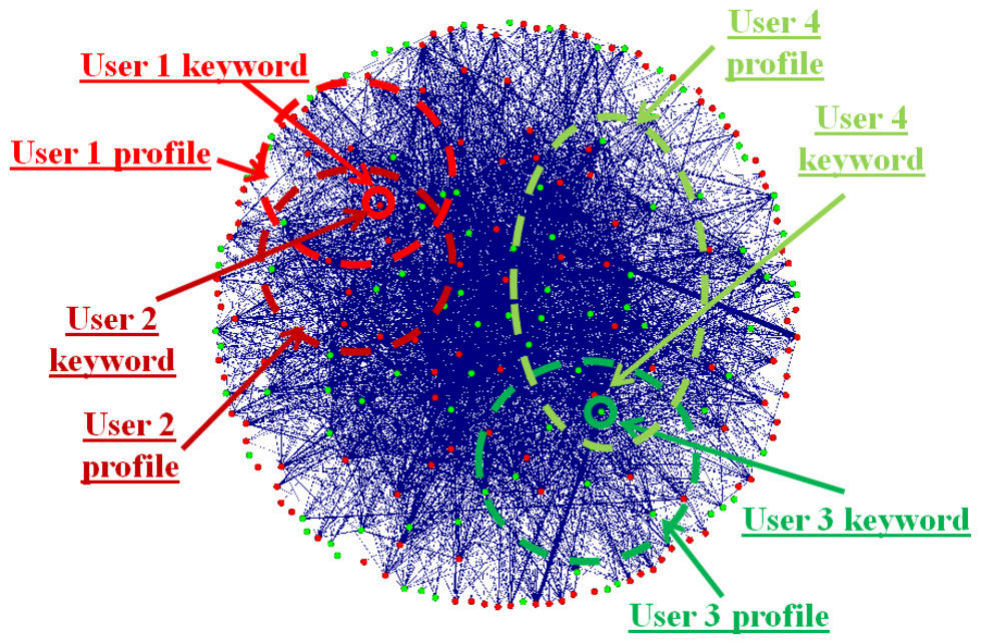
Illustration of the visual exploratory search of semantic relationship graphs. Four users (represented by red and green colors) are considered here.

Once the two sets of domain keywords are available, the semantic relationship graph describing their associations can be inferred via the approaches the above section. The size of the relationship graph could be ranging from dozens of edges to millions of edges. Then, effective and efficient visual exploratory search of these relationship graphs will be a major research issue. The proposed framework for visual exploratory search of these large graphs is illustrated in Figure 5. Overall, the visual exploration procedure will start with user inputting a keyword, and then our VESRGS framework will localize the node of interest to the user, as demonstrated by the colored small circles in Figure 5. In addition, personalized user profiles modeled by graphs (larger dashed shapes in Figure 5) will be used to define sub-graphs from the overall relationship graph by graph matching methods [37]. Thus, a graph of interest (GOI) will be derived for the following detailed visual exploration by a specific user. In this sense, the initial localization of GOI for a user’s visual exploration is obtained by a graph matching procedure. 

Conceptually, there are two major advantages of this graph matching based framework. First, the user’s visual exploration space can be relatively accurately located via the constraint of personalized user model, so that the most relevant information can be provided for users. Therefore, the user does not need to explore the irrelevant search space, which will significantly improve the user’s experience of finding the most relevant information. Second, individual users with different profiles will be able to explore search spaces that are personalized to their own needs. For instance, even user 1 and user 2 input the same keyword (maple syrup), their GOI sub-graphs will be different due to their diverse personal profiles. This method can effectively achieve the goal of personalized search. 

**Figure 6:**
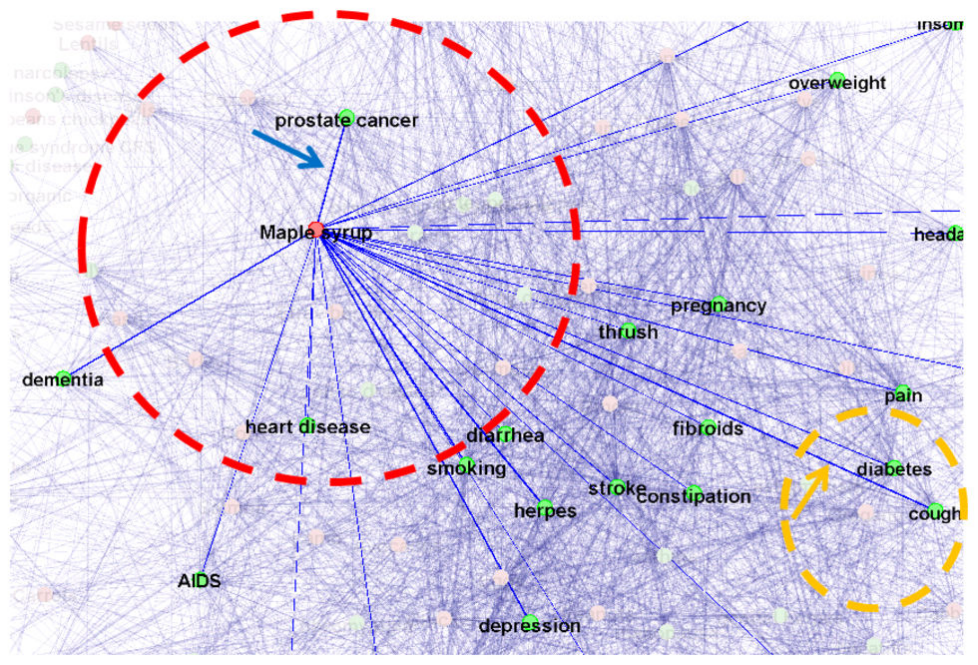
Illustration of visual exploratory search.

Here, we will use user 1 as an example to demonstrate the visual exploratory search procedure. User 1 might continue to explore the human diseases/conditions that are associated with maple syrup within his/her user profile, e.g., the red circle in Figure 6 and read the web links that reported and discussed the relationships between maple syrup and prostate cancer (highlighted by the blue arrow). When the user clicks the links between maple syrup and prostate cancer, our searched links will be prompted out in a separate window for more detailed browsing [47]. This web page explicitly explains the nutrient, particularly zinc, in the maple syrup, and how higher zinc in the prostate can help prevent prostate cancer. The information that maple syrup contains zinc has been confirmed by searching the national nutrient database provided by USDA (http://ndb.nal.usda.gov). 

**Figure 7:**
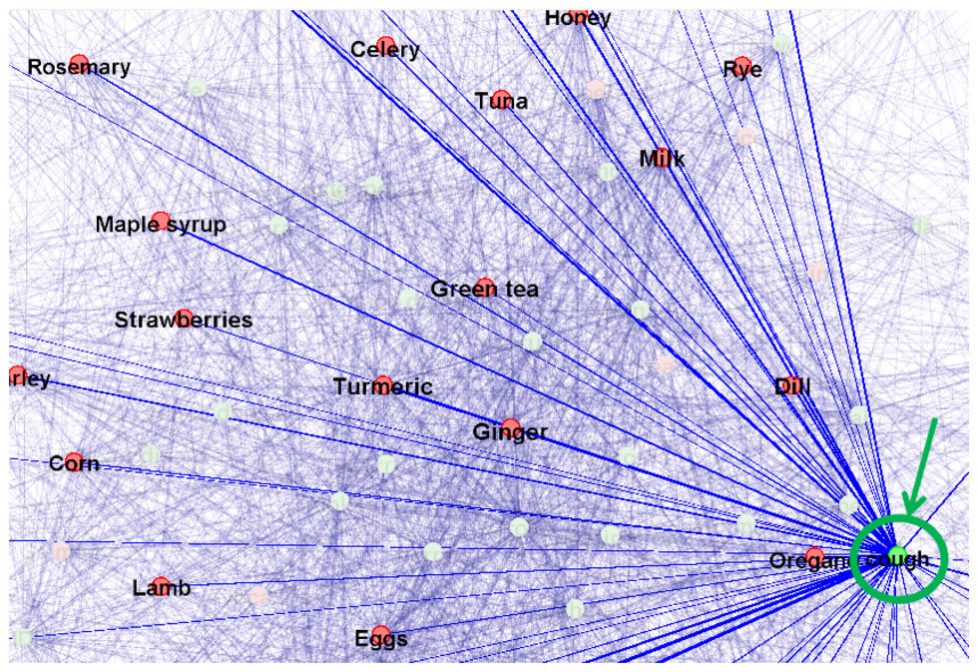
Illustration of the relationship between cough (highlighted in green circle) and other foods.

In addition, user 1 might show interest in the relationship between maple syrup and cough, as illustrated by the orange arrow and dashed circle in Figure 6. Then, the user can shift his/her GOI to the orange dashed circle by easily moving the GOI on the screen or input a new keyword of “cough”. It turns out that multiple websites recommended maple syrup for cough, e.g., one suggested that “recommended a big spoonful of maple syrup just before bedtime for kids with a cough”. Or, user 1 can further explore the relationships, either positive or negative, between cough and other foods, as demonstrated in Figure 7. It has been demonstrated that users are actively involved in the interactive visual exploratory search for learning and discovery according to their personal interests and needs, which is a major advantage of the proposed VESRGS framework. 

### Interactions on smartphones

Although the VESRGS system can run well on typical desktop and laptop machines, as already mentioned in the introduction section, the proposed graph-based visual exploratory search is particularly suitable on smartphones because of the user-friendly interface and ubiquitous Internet connection and portability. In this paper, we use the Android smartphone as a test-bed to demonstrate the smartphone version of the VESRGS framework. 

The interface on Android smartphone is the front-end of this VESRGS framework to the users. It communicates with the cloud server through the ubiquitous mobile Internet to transfer the semantic relationship graphs and query results. Actually, the interaction events from the user will be processed by the cloud server that promptly responds to the changes of the visible GOI on the smartphone. Also, the search results obtained from the cloud server need further post-processing before they are delivered to and displayed on the client smartphone. Since these pre-processing and post-processing steps of semantic relationship graphs need substantial amount of computing resources, the computing capacity of mobile smartphone is currently incapable of handle those computing-intensive steps. Therefore, we move these computing processes to the cloud server. With the powerful computing capacity and high scalability of cloud computing services, the VESRGS framework achieves the real-time processing of GOI graphs. 

**Figure 8:**
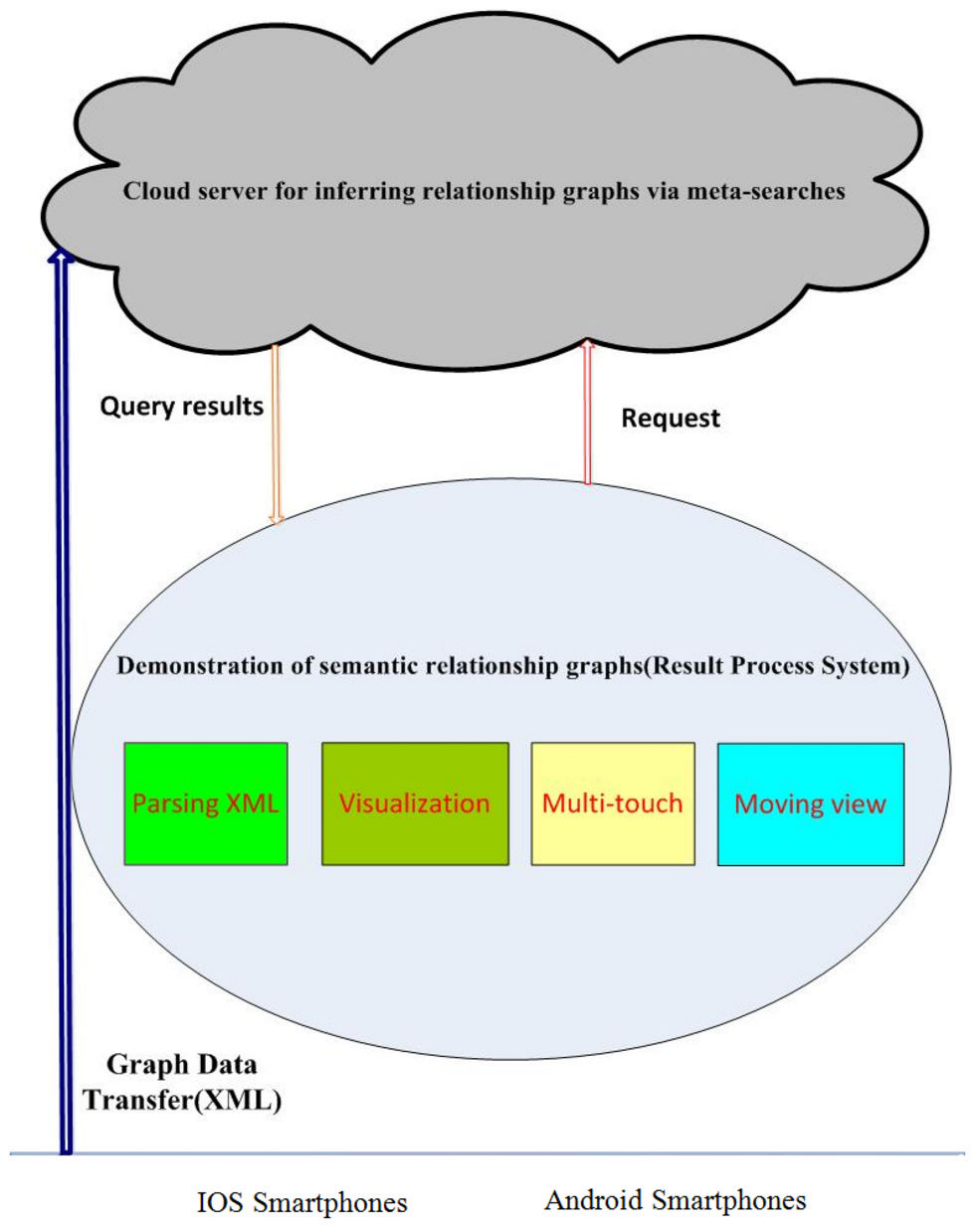
Overview of the smartphone interaction design.

As illustrated in Figure 8, the smartphone interaction system is in the middle layer of the smartphone and the back-end search system. The user interaction procedure is summarized as follows. First, the user inputs a query in the smartphone, and the search system reacts and generates the GOI result. Then, the search system transfers the GOI result to the Result Processing System (RPS). After post-processing steps in the RPS, the system generates an XML data stream and returns them as the search result to the smartphone. In the following paragraphs, we will showcase the whole search process. 

When user 1 enters a keyword query, the server searches the query and generates a GOI sub-graph, denoted as G1. This G1 GOI graph contains an exploratory search result with the highest level of details and the lowest level of abstraction. Due to the limited display capability of smartphone, in comparison with desktop or laptop, the G1 graph needs to be tailored by the RPS for display and interaction on smartphones. The RPS will first group the G1 graph nodes based on the properties of the graph level and the node/edge density, and generate another abstracted graph (G2). This step aims to tailor the GOI graph for multi-scale, or multi-resolution, representation and display on the smartphone. Afterwards, the second step in the RPS is to layout the graphs, include G1 and G2. A desired layout style of the graph will give the user a good experience. The goal of the layout is to build an easily-click graph and generate the graph properties for the follow-up processing. After step 2, the RPS filters G1 and G2, with the query keyword as the center of the GOI result and the smartphone resolution as the filter constraint. The final step in RPS is to generate the XML data stream, represents the GOI graph, and transmits it to the smartphone. Finally, the smartphone receives the XML data and display it to the user. 

**Figure 9:**
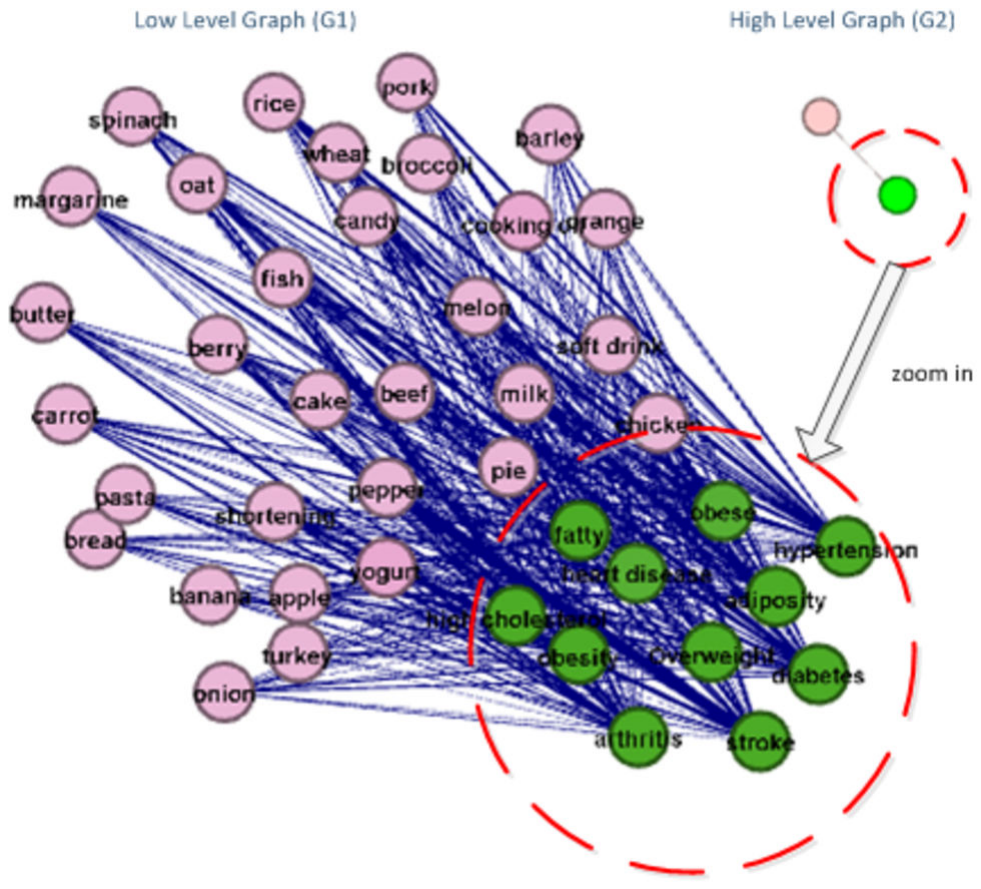
Example of multi-scale GOI graphs.

From the user interaction perspective, the user has four main types of actions on the smartphone: zoom, drag, click and filter, which will be detailed as follows. In the beginning, the graph displayed on the smartphone is G2. It contains a high-level, abstracted view of the GOI. The node in this graph represents a group of lower level nodes. For example, the GOI graph in Figure 9 is the result when a user searched “fatty” and “chicken”. The search result will first be displayed as high-level view on the right side. When user 1 zooms in the graph, it will show more and more detailed results with denser graph nodes. This coarser-to-finer visualization approach has been widely used in the data visualization field. By this approach, users first see a globe view of the semantic relationship graph. If the user wants to know more about specific graph node, he/she can zoom in the graph, browse, and explore. This procedure is very similar to those in the above sections. For instance, when the user wants to change the focused area, he/she can drag the graph. If there is no more data within the area when dragging to the edge in the data cache, the smartphone client will automatically retrieve more data from the server. 

To facilitate the user’s visual exploratory search, the semantic relationship graph G1 and G2 will be classified into different categories of sub-graphs based on the knowledge space, and will be marked with different colors. The user can filter the result by matching his/her personal profile with the GOI and taking the intersection (Figure 5), thus offering the user a good view of what he/she is most interested in. If the user has found the exact answer of his/her query or he/she has found something that he/she wants to know more about the details, the user can click the edge on the GOI graph and obtain the web pages in a separate web browser. If the user wants to continue the visual exploration process, he/she can return to the GOI graph view. Notably, the click history could be used for personalized user profile construction if the user agrees.

In summary, the smartphone interaction interface offers a novel, effective way for users to perform visual exploratory search of semantic relationship graphs. We premise that the user’s experience of visual exploration of relationship graphs on smartphones will be more natural, engaging, interactive, enjoyable and productive, than that on traditional desktop or laptop machines. This premise will be evaluated in the next section of experimental results. 

## Results and Discussion

We designed and conducted four sets of experiments to evaluate the proposed VESRGS framework for relationship graph inference, the approaches of visual exploratory search, and the interface of interactions on smartphones, respectively. 

### Evaluation of inferred relationship graphs

In this experiment, we examined two test-bed relationships including those between selected foods and obesity-related conditions and those between selected foods and cardiovascular diseases. Table 1 and Table 2 summarize the selected terms (term space A and B in Figure 2) in these two applications. Both of these two sets of terms were provided by an expert in food sciences and confirmed by another expert in biomedical health sciences. The meta-search and graph inference approach were them applied to infer the relationship graphs from these two sets of terms. Table 3 and Table 4 show the strongest associations in these two experiments. It turns out that all of the inferred food/health associations (labeled as positive, negative or neutral in the last columns of Tables 3-4) are reasonable, given current food/health science domain knowledge [48]. An expert in food sciences has confirmed the results in Tables 3-4. 

**Table 1:** List of query terms of selected foods (in green) and obesity related issues (in red).

Terms for obesity	Overweight, obesity, obese, fatty, adiposity, diabetes, hypertension, high cholesterol, stroke, heart disease, and arthritis.
Terms for foods	milk, yogurt and cheese, cooking oil, butter, margarine and shortening, apples, oranges, bananas, berries and melons, wheat, rice, oats, barley, bread and pasta, chicken, fish, turkey, pork and beef, candy, soft drinks, cake, pie and ice cream, spinach, carrots, onions, peppers, and broccoli.

**Table 2:** List of query terms of selected foods (in green) and cardiovascular related issues (in red).

Terms for cardiovascular disease	Heart disease, angina, aortic dissection, aortic stenosis, arrhythmia, atrial fibrillation, blood clots, cardiomyopathy, chest pain, laudication, congenital heart disease, congestive heart failure, deep vein thrombosis, edema, endocarditis, fainting, fitness, heart attack, …
Terms for foods	sweet potato, Green leafy vegetable, Potherb, green vegetable, greens, leafy green, salad green, carrot, broccoli, pumpkin, squash, chicken breast, turkey breast, tomato sauces, pasta, onions, garlic, pizza, low-salt, peanut, walnut, almond, olive oil, canola oil, salmon, mackerel, sardines, herring, skim milk, fat free milk, oatmeal, shredded wheat, low-no sugar added cereal, whole wheat bread, fruit, apple, orange, black grape, red grape, grape juice, grape, grapefruit, dried fruit, apricots, dates, prunes, cantaloupe, yogurt, fat free yogurt, …

**Table 3:** Top 10 strongest associations in experiments of obesity.

Terms for obesity	Terms for food	weight
stroke	pepper	2098 (positive)
adiposity	milk	1286 (neutral)
obese	candy	946 (negative)
diabetes	oat	880 (positive)
hypertension	rice	868 (positive)
obesity	yogurt	846 (positive)
arthritis	spinach	803 (positive)
obese	milk	643 (neutral)
arthritis	butter	633 (positive)
hypertension	pork	614 (negative)

**Table 4:** Top 10 strongest associations in the experiments of cardiovascular diseases.

Terms for cardiovascular diseases	Terms for food	weight
palpitations	garlic	1910 (positive)
edema	salt	1748 (negative)
angina	chips	1689 (negative)
angina	sauces	1645 (negative)
atrial fibrillation	cheese	1562 (negative)
heart attack	garlic	1531 (positive)
atrial fibrillation	pasta	1457 (positive)
atrial fibrillation	sugar	1424 (negative)
myocarditis	grapefruit	1390 (negative)
palpitations	salt	1337 (negative)

**Figure 10:**
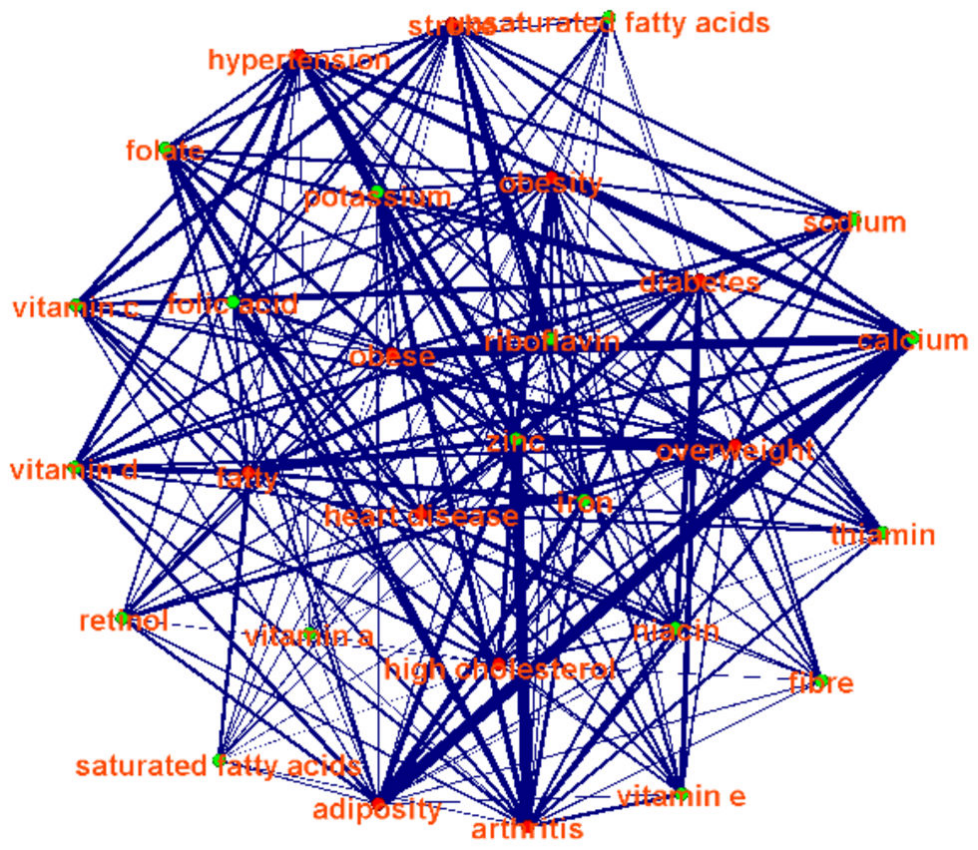
Example of associations between nutrients (green) and health conditions (red). The edge width represents strength.

As the role of nutrients is the basis for explanation of the health effect of a certain food item, we conducted nutrient component analysis of the food items in Table 1, and correlated the components to the effects of health enhancement/disease involvements. Specifically, the nutrients components of food items were obtained from the National Nutrient Database provided by USDA [49], and we performed a pilot study of the associations between the nutrients of fiber, calcium, iron, sodium, zinc, potassium, vitamin C, thiamin, riboflavin, niacin, folate, folic acid, vitamin A, retinol, vitamin E, vitamin D, saturated fatty acids, unsaturated fatty acids and the health conditions in Table 1. It turns out that the results are quite reasonable, as shown in Figure 10. These results suggest that the methods are effective in inferring widely reported associations between foods and health conditions. Importantly, our graph inference approach summarized these most frequently reported associations systematically and comprehensively. 

In addition, we measured the graph properties of these relationship networks via graph theoretic metrics such as average edge degree and edge weight. Here, only association edges that were reported by over 50 websites are kept in the relationship graph. It is found that the average edge degree is pretty large (80), suggesting that there are strong interactions among foods and human disease or conditions. Also, the average edge weight is (978), demonstrated that these association between foods and diseases/conditions are widely reported on websites. 

**Figure 11:**
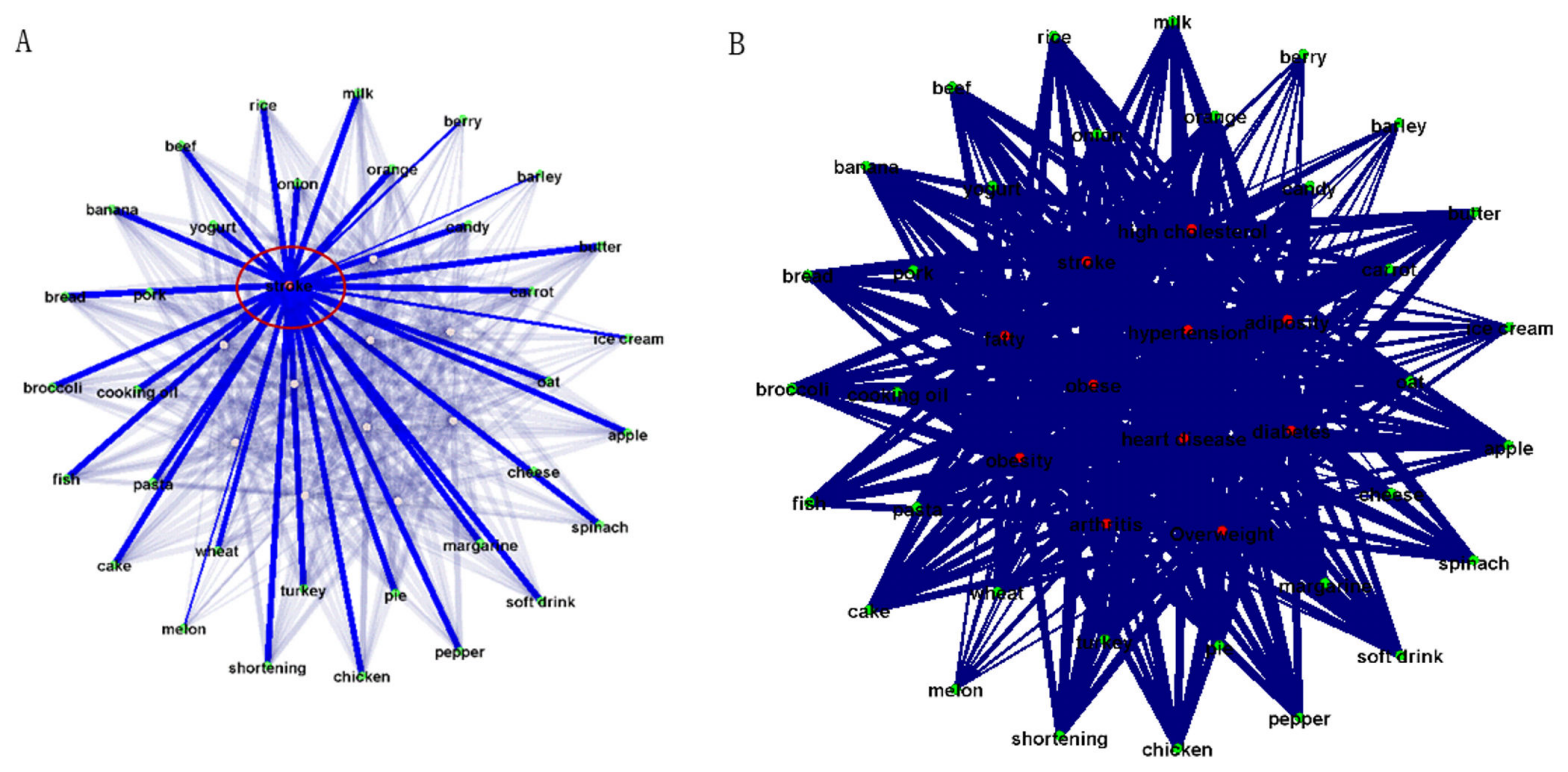
Comparison between the used co-occurrence method and latent semantic analysis (LSA) method. The keyword “stroke” is used as an example here. (a) The result for “stroke” by LSA method. (b) The whole graph inferred by LSA method.

We have compared the relationship graph inference method based on keyword co-occurrences with the latent semantics analysis (LSA) method [50]. The Stanford Infomap NLP software package [51] was used for LSA. Briefly, the Infomap software is a variant of LSA on free-text corpora that learns vectors representing the meanings of words in a vector-space. It indexes the documents in the corpora it processes, and can perform word-word semantic similarity computations using the learned model. Figure 4 and Figure 11a show examples of the relationship graphs for “stroke” by the co-occurrence method and by Infomap LSA. It is evident that the LSA-derived graph has very similar or equal edge strength between the keyword “stroke” and many other food links, suggesting that LSA tends to smooth out the intrinsic differences between the edge strengths, and generates relative uniform edges. In comparison, the used co-occurrence method used in this paper generates quite meaningful connection patterns between diseases and foods, as demonstrated by using the keyword stroke as an example in Figure 4. Figure 11b shows all of the edges for the testing dataset in Table 1. It is evident that most of the edges tend to have similar or uniform connection strengths, which may contain many false positives. Instead, the co-occurrence based method can generate more distinctive patterns of edge strengths (e.g., Figure 4) that are meaningful according to domain knowledge. 

### Evaluation of visual exploratory search

**Figure 12:**
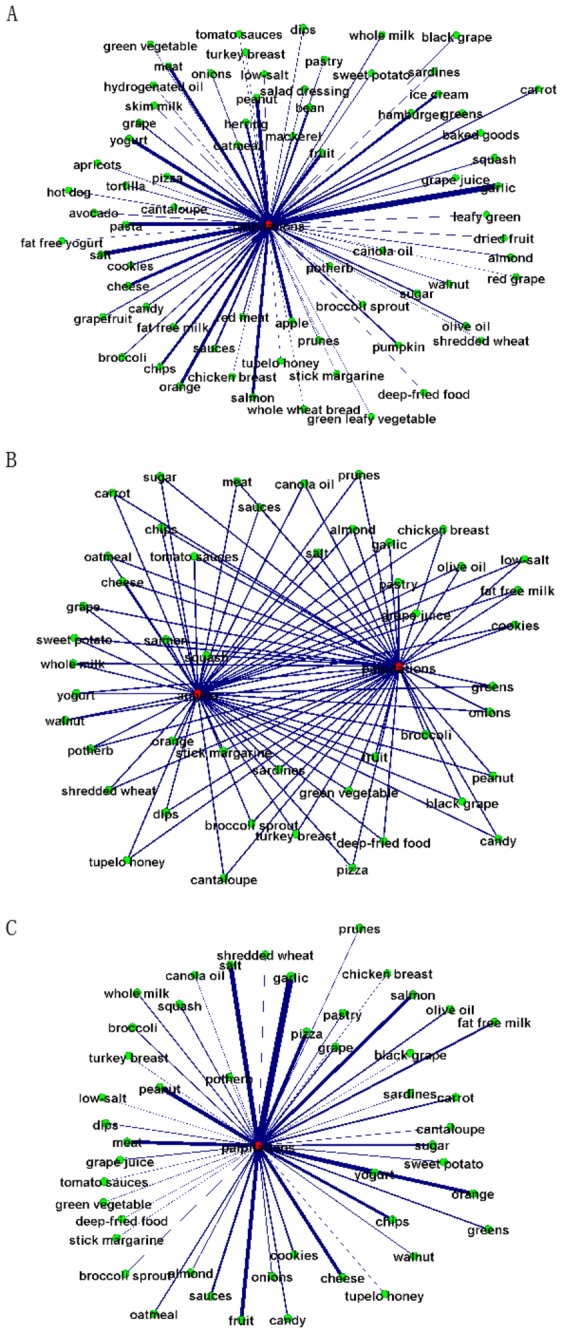
(a) GOI without user profiles; (b) A user profile modeled by a graph; (c) Intersection of graphs in (a) and (b).

Here we use an example to demonstrate how personalized search is achieved by integrating user profile models into the graph matching procedure illustrated in Figure 5. We evaluated eight users who were interested in “palpitations”. For the keyword “palpitations”, the same GOI was generated for all of the eight users without using user profiles. The GOI contains 74 nodes and 73 edges, as shown in Figure 12a. If we integrate a personalized user profile that is composed of a graph with 50 nodes and 96 edges (shown in Figure 12b), the intersection of GOI and the user model graph results in a new personalized GOI with 49 nodes and 48 edges, as shown in Figure 12c. We tested the eight different users and it was found that the intersection overlaps of the GOI and eight user model graphs are 65.8%, 64.4%, 72.6%, 71.2%, 69.9%, 61.6%, 56.2%, 67.1%, respectively. This result suggests that it is critical to integrate the user’s personal preferences and profiles into the individualized exploratory search of relationship graphs. 

### Evaluation of interactions on smartphones

**Figure 13:**
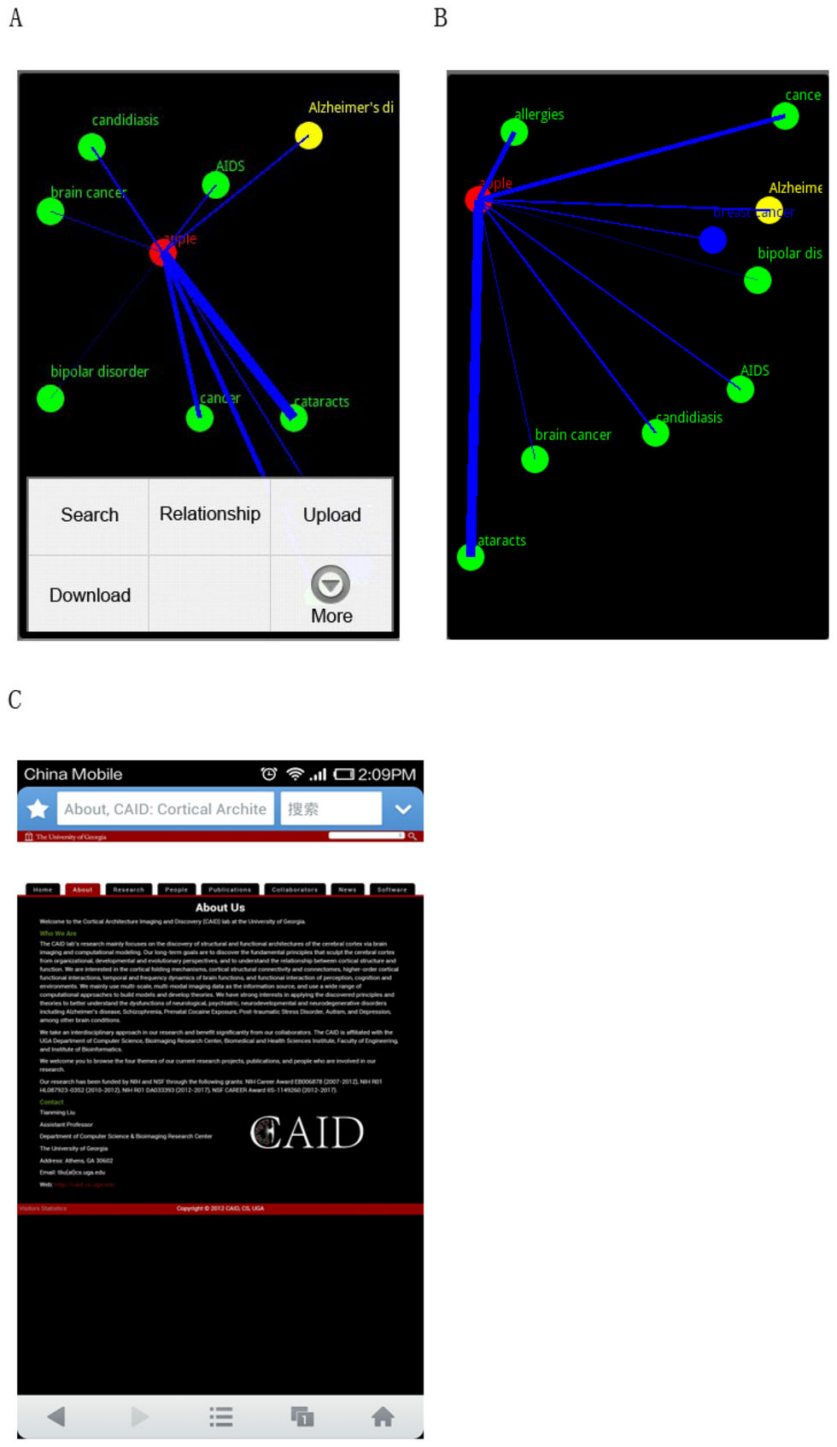
(a)-(b). Multi-scale views of GOI and interactions, (c) an example of web page on a GOI edge.

This experiment of interactions on smartphones is based on the data obtained in the above sections. The users used the VESRGS framework by beginning with entering keywords. The resulted GOI graph returned from the server is described as an XML data stream. The returned GOIs were represented by multi-scale graphs, and the user can then navigate through these multiple scales (e.g., Figures 13a-13b). For a better user experience, the GOI edges were represented by curve shapes, and the color of the edge curve is the mixed color of the two end nodes’ colors. 

When performing the visual exploratory search, the user can drag, zoom, and/or navigate the GOI graphs (e.g., Figure 13b). The graph data of other areas out of the visualization focus has been cached to facilitate the visual exploration. In this way, the user can explore large areas and learn/discover a wide range of food/health relationships. When the user clicks the GOI edge, which represents a URL containing both of the keywords at the end of the edge, the Android smartphone client then pops up the web page and displays the content to the user (Figure 13c). After the user finishes reading the web page, he/she can return to the GOI by clicking the return button on Android smartphone. All of the functions have been realized in the interactions on Android smartphones and the tester users’ feedback has been excellent. 

### User evaluations

To quantitatively evaluate the proposed VESRGS framework, we employed subjective user evaluation which aimed to measure the user’s experience of using our approach versus using other popular approaches such as Google, Yahoo!, and Bing. The usability of finding the most relevant food/health relationship was used as the major evaluation metric. The evaluation scales are classified as: excellent, very good, good, fair and poor. For the five-item score, it is expressed as a single number in the range of 5 to 1, where 1 is the lowest perceived quality and 5 is the highest perceived quality. We invited ten volunteers to survey these search methods. In the survey, the test-beds were based on the keywords search results in Tables 1-2. The average scores of the survey results from ten participants were calculated, and the results for VESRGS, Google, Yahoo!, and Bing are 4.6, 3.54, 3.54, and 3.536, respectively. It turns out that the proposed VESRGS framework is substantially better than other popular systems in terms of finding the most relevant relationships.

## Conclusions

In summary, the proposed VESRGS framework is based on three key components including inference of relationship graphs via meta-search, visual exploratory search on relationship graphs, and user interaction on smartphones. Our experiments have demonstrated that the VESRGS framework possess superiority in comparison with other search approaches in exploring relationships among interacting domains such as food and health conditions. It has been shown that relationship-based search has advantages over traditional keywords-based lookup search in discovering intrinsic associations among large-scale concepts and semantic networks. Personalized search can be naturally formulated as a graph matching problem in this relationship-based search methodology, which is considered as a major advantage in comparison with other exploratory search [1-9] and navigational faceted search [52] approaches. Therefore, the VESRGS framework is particularly suitable for exploratory, semantics-based, and personalized search on the mobile Internet. 

There are several lines of directions for future improvements and consolidations of the VESRGS framework. First, the discovered semantic relationships between two domains of terms need to be further differentiated in the future, e.g., whether or not they exhibit causal relationships need to be determined. However, the full elucidation of these causal relationships entail carefully designed biomedical and behavior experiments by domain experts. For instance, the relationship between milk and children obesity has been complex and its elucidation needs significant efforts of experiments [48]. Also, performing meta-searches in additional professional healthcare search engines such as PubMed, Healthline, Healthfinder and WebMD could potentially contribute to the VESRGS framework for food/health applications. Second, the experiments in this paper so far are limited to the relationships between food and health domains. One of our ongoing works is to investigate the relationships between other domains such as the associations between environmental factors and human diseases and conditions. Third, in current stage, the user visual exploratory search is constrained to the relationship graphs inferred off-line based on a prior domain knowledge. In the future, we plan to develop real-time inference of relationship graphs for online visual exploratory search services to users, when users input keywords that do not exist in our off-line relationship graphs. In this scenario, the powerful cloud computing capability will play important roles. 

Finally, we believe that relationship-based exploratory search has key advantages over traditional keyword-based lookup search in a variety of ways including rich semantics, learning and discovering, more relevance, personalization, and natural interaction. The semantic graphs of relationships among domain terms were inferred from large-scale meta-search result, which differentiates our approach from the RDF-based Linked Data [53]. In addition to the natural language processing field that uses relationships to represent semantics such as in WordNet [20,21], many other disciplines have used inter-relationships to represent semantics and functional meanings. For instance, in the computational neuroscience field, researchers have used the functional connectivity and interaction to represent semantics in the human brain [54]. In computational systems biology, people have used inter-relationships among genes and proteins to represent biological events [55]. In the webpage browsing field, relation browsers [56] were proposed to help people explore the relationships across different attribute sets, thus enabling understanding the scope and extent of the corpus. Thus, we conjecture that relationships among domain terms could be a generic approach of representing semantics and meanings, and we envision that this new school of relationship-based search could significantly facilitate both domain experts and regular users when they are exploring mountains of data on the Web and seeking the most relevant information/knowledge for themselves. 
